# Preparation, Characterization and Sensitive Gas Sensing of Conductive Core-sheath TiO_2_-PEDOT Nanocables

**DOI:** 10.3390/s90906752

**Published:** 2009-08-27

**Authors:** Ying Wang, Wenzhao Jia, Timothy Strout, Yu Ding, Yu Lei

**Affiliations:** Department of Chemical, Materials and Biomolecular Engineering, University of Connecticut, 191 Auditorium Road, Storrs, CT 06269, USA; E-Mails: ywang@engr.uconn.edu (Y.W.); wenzhao.jia@engr.uconn.edu (W.J.); tim.strout@huskymail.uconn.edu (T.S.); yud08001@engr.uconn.edu (Y.D.)

**Keywords:** poly(3,4-ethylenedioxythiophene), nanocables, gas sensor, vapor phase polymerization, electrospinning

## Abstract

Conductive core-sheath TiO_2_-PEDOT nanocables were prepared using electrospun TiO_2_ nanofibers as template, followed by vapor phase polymerization of EDOT. Various techniques were employed to characterize the sample. The results reveal that the TiO_2_ core has an average diameter of ∼78 nm while the PEDOT sheath has a uniform thickness of ∼6 nm. The as-prepared TiO_2_-PEDOT nanocables display a fast and reversible response to gaseous NO_2_ and NH_3_ with a limit of detection as low as 7 ppb and 675 ppb (S/N=3), respectively. This study provides a route for the synthesis of conductive nanostructures which show excellent performance for sensing applications.

## 1. Introduction

Controllable synthesis of conducting polymer nanostructures has been intensively investigated in the past decades due to their unique optical and electronic properties. Among various methods in the synthesis of conducting polymer nanostructures, the template method is the most efficient and controllable one [1,2]. Various templates such as carbon nanotubes and anodic alumina oxides have been used to provide scaffold/support or spatial confinement for the growth of desired conducting polymers [3–5]. Recently, electrospun nanofibers have emerged as ideal supporting templates in the fabrication of core-sheath nanostructures with a narrow diameter distribution because the deposition of conducting polymers on electrospun nanofibrous template could potentially yield a class of nanomaterials with a large surface to volume ratio, highly porous structure, and an ultrathin conducting layer, which favor the gas adsorption/desorption process and thus make such materials attractive as potential sensing elements [6,7].

It has been well documented that conducting polymers are sensitive to environmental compositions [8]. Among the commonly used conducting polymers, poly(3,4-ethylenedioxythiophene) (PEDOT) has attracted a lot of attention in recent years due to its excellent properties such as long-term stability, high conductivity, optical transparency in its doped state, low band gap, and moderate redox potential [9–12]. PEDOT is commonly accepted to be more environmentally stable than other conducting polymers such as polypyrrole and polyaniline [13,14]. These features make PEDOT and its derivatives appealing in gas sensing. To date, numerous PEDOT based gas sensors have been proposed to detect gaseous analytes such as NH_3_ [15–19], HCl [15,16], alcohol [20,21], and NO [22]. To synthesize PEDOT for such applications, electrochemical polymerization and chemical polymerization are widely used. Compared with electrochemical polymerization, chemical polymerization of EDOT can provide uniform coating on both conducting and non-conducting surface, requires relatively simple equipment, and adapts a flexible procedure; therefore, it has been widely employed to deposit PEDOT on different substrate [1,23].

Recently, a conducting polymer such as polypyrrole has been successfully coated on nanofbers and its application for ultrasensitive NH_3_ detection has also been demonstrated [6,24]. Herein, we report a facile method to fabricate conductive core-sheath TiO_2_-PEDOT nanocables by vapor phase polymerization of EDOT. Electrospun TiO_2_ nanofibers were used as the template. Various characterization techniques were applied to characterize the as-prepared samples and the results reveal that the TiO_2_ core has an average diameter of ∼78 nm, while the PEDOT sheath has a uniform thickness of ∼6 nm. We further demonstrated the application of the TiO_2_-PEDOT nanocables for NO_2_ and NH_3_ detection. The as-fabricated sensor exhibits fast response and good recovery upon exposure of gaseous analytes and also shows excellent limit of detection. This study provides a simple strategy to fabricate conductive nanomaterial, which is promising in various sensing applications.

## 2. Results and Discussion

### 2.1. Characterization of Conductive Core-sheath TiO_2_-PEDOT Nanocables

**Figure 1.**
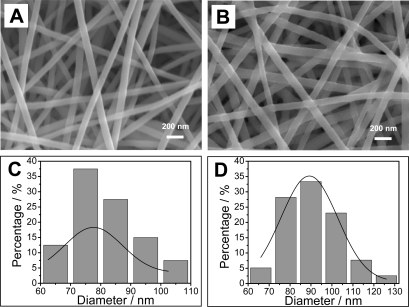
(A) SEM image of electrospun TiO_2_ nanofibers. (B) SEM image of TiO_2_-PEDOT nanocables after the vapor phase polymerization of PEDOT on TiO_2_ nanofibers. (C) and (D) Histograms showing the size distribution of TiO_2_ nanofibers and TiO_2_-PEDOT nanocables, respectively.

Electrospinning is a straightforward and versatile technique to produce continuous fibers with nanoscale diameter and a large surface to volume ratio [25]. In this research, TiO_2_ is chosen as template in the preparation of conductive TiO_2_-PEDOT nanocables due to its excellent chemical stability in oxidizing environments. Figure 1A shows the typical SEM image of the as-prepared TiO_2_ nanofibers. It is clearly seen that the sample consists of numerous randomly-oriented nanofibers with relatively uniform morphology and size in the area examined. When the white TiO_2_ nanofibers were dipped into FeCl_3_-ethanol solution and dried in air, their color became yellowish due to the adsorption of FeCl_3_. After the sample was exposed to EDOT vapor at 95 °C [26], the adsorbed FeCl_3_ triggered the vapor phase polymerization of EDOT. One obvious phenomenon is that the sample became dark blue, indicating the formation of PEDOT on the surface of TiO_2_ nanofibers. Figure 1B reveals that the morphology of the as-prepared conductive TiO_2_-PEDOT nanocables is similar to that of TiO_2_ nanofibers and only the diameter is slightly bigger than that of corresponding TiO_2_ nanofibers. Diameter distributions for the samples before and after PEDOT coating were further obtained using 40 randomly chosen TiO_2_ nanofibers and TiO_2_-PEDOT nanocables in corresponding SEM images and then analyzed by Gaussian fitting. One can see from Figures 1C-D that more than 85% of the TiO_2_-PEDOT nanocables have diameters ranging from 72 to 108 nm with a Gaussian mean diameter of 89.2 nm, while the diameter of TiO_2_ nanofiber template has a Gaussian mean value of 77.6 nm. As the difference of the two Gaussian mean diameters is 11.6 nm, which is equal to twice of the thickness of PEDOT sheath, the thickness of the coated PEDOT layer is calculated to be ∼5.8 nm.

**Figure 2.**
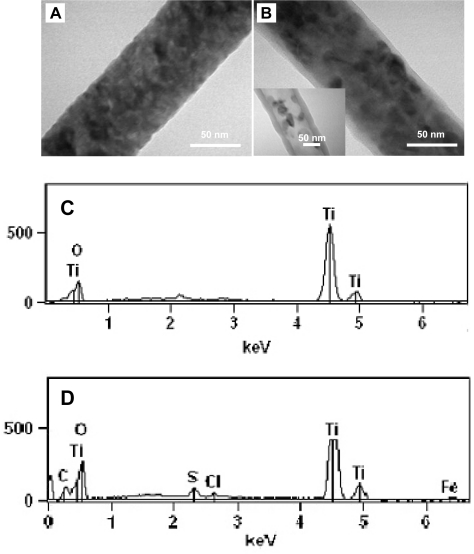
(A) TEM image of an individual TiO_2_ nanofiber. (B) TEM image of a core-sheath TiO_2_-PEDOT nanocable. The inset shows PEDOT nanotube obtained by selectively removing TiO_2_ core in 5 mol/L HF aqueous solution. (C) EDX element analysis result for TiO_2_ nanofibers. (D) EDX element analysis result for TiO_2_-PEDOT nanocables.

The detailed morphology of the samples were further characterized and analyzed by TEM. Figure 2A and B show typical TEM images of the TiO_2_ nanofiber before and after PEDOT-coating. Clearly seen from the TEM images, TiO_2_ nanofiber has a rough surface and consists of many nanoparticles (Figure 2A), while TiO_2_-PEDOT nanocable displays a core-sheath structure with an extremely thin (∼6 nm) layer of PEDOT (Figure 2B), which is in good agreement with the analysis of Gaussian fitting previously. The TEM images also clearly indicate the uniformity of TiO_2_ core as well as the uniformity and continuity of PEDOT sheath. In addition, the vapor polymerization of EDOT using FeCl_3_ as an oxidant would not transform the morphology of the electrospun TiO_2_ nanofibers. It has been reported that TiO_2_ could be dissolved in HF solution [27]. In order to better reveal the core-sheath structure after PEDOT-coating, the as-prepared TiO_2_-PEDOT nanocables were soaked into a 5 mol/L HF solution under overnight-sonication to selectively remove TiO_2_ cores. It can be seen that a tubular structure was indeed observed after the dissolution of TiO_2_ cores. However, the PEDOT layer after HF treatment is thicker than that before treatment (inset of Figure 2B). A similar phenomenon has been observed by other group [28] and it may be attributed to the irreversible swelling-effect of PEDOT in solution. Compared to conventional PEDOT films prepared by chemical polymerization [29,30], the as-prepared PEDOT layer is much thinner, which may be attributed to the limited adsorption of oxidant FeCl_3_ on TiO_2_ surface. Such ultrathin PEDOT layer makes it promising in various sensor applications. The composition change during the PEDOT-coating process could also be revealed by EDX analysis. Figure 2C reveals that the TiO_2_ nanofibers are only composed of elements of titanium and oxygen. No other peak was observed, indicating that the PVP used in the electrospinning has been completely removed by calcination. The EDX peaks were further fitted by Chi-squared filter applying the Proza (Phi-Rho-Z) correction method, and the calculated ratio of Ti to O was an approximate 1:2, in good agreement with the stoichiometric proportion of TiO_2_. In contrast, peaks associated with carbon and sulfur were observed for the sample after PEDOT-coating (Figure 2D), indicating the formation of PEDOT. The iron and chlorine were also identified in the PEDOT-coated sample, which was reasonable as FeCl_3_ was used as the oxidant and Fe and Cl were left in the PEDOT layer after the vapor phase polymerization.

**Figure 3.**
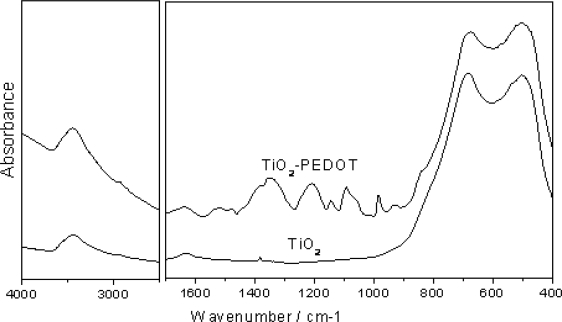
FTIR spectra of the electrospun TiO_2_ nanofibers and TiO_2_-PEDOT nanocables.

The formation of PEDOT on TiO_2_ was further confirmed by the FTIR spectra (Figure 3). The TiO_2_ nanofibers have the main bands between 400 and 700 cm^−1^, which are attributed to Ti–O stretching and Ti–O–Ti bridging stretching modes [31]. In addition, no band related to CH stretching of hydrocarbon was observed, indicating that PVP has been removed by calcination at 500 °C. After PEDOT-coating, the characteristic bands corresponding to PEDOT were observed in the range of 900–1520 cm^−1^. Specifically, the peaks at around 1,520 and 1,344 cm^−1^ can be assigned to C=C and C–C stretching mode of thiophene ring, respectively, while the peaks centered at 1,208, 1,145, and 1,093 cm^−1^ are ascribed to C–O–C bond stretching in the ethylene dioxy group. The peaks at 985 and 834 cm^−1^ can be assigned to C–S bond in the thiophene ring. It has also been reported that C–S bond usually have another peak at ∼682 cm^−1^. However, this peak was not observed in FTIR spectrum of TiO_2_-PEDOT, and it may be concealed by the intense TiO_2_ peak in the same region. The presence of water and hydroxy groups was also manifested, as the existence of a bending mode of H–O–H at 1,635 cm^−1^ and a strong stretching vibration of O–H at 3,440 cm^−1^, which can be attributed to the moisture bound to the samples and/or the possible presence of reduced oxidant salt species FeCl_2_ in a hydrate form [6]. Both IR spectra of TiO_2_-PEDOT nanocables and TiO_2_ nanofibers were in good agreement with the literature [26,31–33]. It is noteworthy that the strong TiO_2_ peaks in the region between 400–700 cm^−1^ are observed for both samples and the PEDOT coating cannot conceal their presence, indicating that PEDOT sheath is ultra-thin, in good agreement with TEM results.

**Figure 4.**
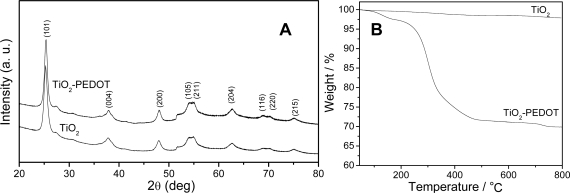
(A) XRD patterns of the electrospun TiO_2_ nanofibers and TiO_2_-PEDOT nanocables. (B) TGA in the oxygen atmosphere of TiO_2_ nanofibers and TiO_2_-PEDOT nanocables.

Figure 4A shows the XRD patterns of the TiO_2_ nanofibers and the core-sheath TiO_2_-PEDOT nanocables. It was found that TiO_2_ nanofibers are crystalline and all diffraction peaks can be assigned to the anatase phase of titania, in good agreement with other reports [6,25]. The similar XRD pattern was also obtained for TiO_2_-PEDOT nanocables, implying that PEDOT layer is ultra-thin and likely to be in an amorphous state. In order to study the thermal stability of PEDOT coated on TiO_2_ template, the thermogravimetric (TG) analysis was also carried out before and after PEDOT-coating and presented in Figure 4B. The TG of TiO_2_-PEDOT nanocables shows an initial weight loss of 2.6% is observed up to 180 °C, which may be attributed to the evaporation of the physically adsorbed water and the leaching of the dopant from the polymer composite matrix. The largest weight loss (25.8%) occurs from 180 °C to 505 °C, which is due to the degradation of PEDOT. A total weight loss of 28.4% has been observed up to 800 °C. As a comparison, the thermal behavior of the TiO_2_ nanofibers is very stable. There is no significant weight loss observed in the temperature range from room temperature to 800 °C, which is likely due to the highly thermal stability of TiO_2_.

### 2.2. Gas Sensing Performance

TiO_2_ nanofibers are very fragile. However, the PEDOT coating on TiO_2_ nanofibers enhances the mechanical property of the as-prepared TiO_2_-PEDOT nanocables. The TiO_2_-PEDOT nancables possesses an ultra-thin layer of PEDOT, porous mesh structure, and highly specific surface to volume ratio, which favor the adsorption and desorption of gas molecules. In addition, SEM and TEM images show that the ultra-thin PEDOT layer is homogeneous and uniform, thus it can greatly suppress the noises generated from structural defects and then have the potential to improve the limit of detection [3]. Moreover, the PEDOT layer on the TiO_2_ nanofibers has a longer conjugation length which can facilitate the charge transport in the TiO_2_-PEDOT nanocables [3]. All these features make the as-prepared core-sheath TiO_2_-PEDOT nanocables an excellent sensing material.

**Figure 5.**
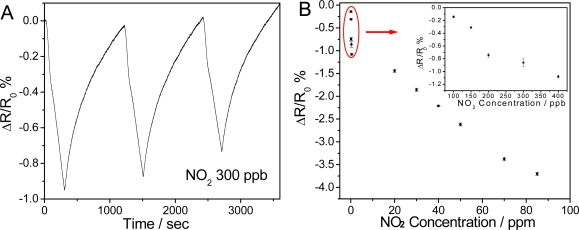
(A) Typical response of TiO_2_-PEDOT nanocables upon periodic exposure of 300 ppb NO_2_. (B) The calibration plot for NO_2_ at an applied DC bias of 0.1 V and room temperature.

**Figure 6.**
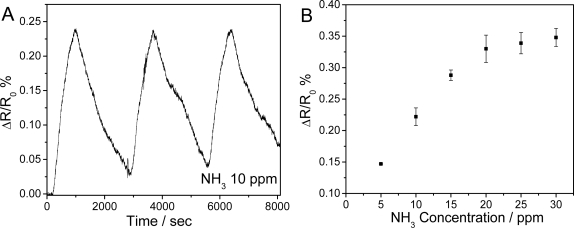
(A) Typical response of TiO_2_-PEDOT nanocables upon periodic exposure of 10 ppm NH_3_. (B) The calibration plot for NH_3_ at an applied DC bias of 0.1 V and room temperature.

Figures 5A and 6A represent typical electrical responses of the TiO_2_-PEDOT nanocables as a function of time upon periodic exposure to gaseous NO_2_ (300 ppb) and NH_3_ (10 ppm), respectively. Air was chosen as the carrier gas in both experiments in order to simulate the most common sensing environment. Upon the exposure of TiO_2_-PEDOT nanocables to 300 ppb NO_2_, the normalized resistance of TiO_2_-PEDOT nanocables decreases rapidly by 0.861%. On the contrary, exposure of the same device to 10 ppm NH_3_ efficiently increases the resistance by 0.222%. For three “on-off” cycles of NO_2_ or NH_3_, the TiO_2_-PEDOT nanocables show fast, reproducible, and sensitive responses for all three cycles, suggesting that TiO_2_-PEDOT nanocable is a good sensing element in the detection of NO_2_ and NH_3_. By purging with dry air, the response of TiO_2_-PEDOT nanocables can be near-completely recovered, indicating the quick desorption of gas molecules from the ultra-thin PEDOT layer and the good reproducibility of TiO_2_-PEDOT nanocables based sensor. The good performance and fast response/recovery can be attributed to the highly porous structure and ultrathin PEDOT-coating layer (∼6 nm), in which porous structure allows the free access of analyte molecules to PEDOT while ultrathin PEDOT layer greatly reduces the diffusion resistance.

The gas sensing mechanism of TiO_2_-PEDOT nanocables can be illustrated based on the electrical properties of the sensing element and the chemical property of the analyte molecules. In this study, FeCl_3_ was used as oxidant and the as-prepared PEDOT is *p*-type. NO_2_ has an unpaired electron and is known as a strong oxidizer. Upon NO_2_ adsorption, charge transfer from PEDOT surface to NO_2_ is likely to occur because of the strong electron-withdrawing power of the NO_2_ molecules, which might generate more hole-carriers in PEDOT and thus result in the enhanced conductance of PEDOT. In contrast, NH_3_ is a Lewis base with a lone electron pair that can be donated to other species. The charge transfer between NH_3_ and *p*-type PEDOT leads to the formation of neutral polymer chains and results in the decrease of charge carrier density, which decreases the conductivity of PEDOT.

The response of TiO_2_-PEDOT nanocables as a function of NO_2_ and NH_3_ concentration was presented in Figures 5B and 6B, respectively. For both NO_2_ and NH_3_, the response of TiO_2_-PEDOT nanocables increases with the analyte concentration. At low NO_2_ concentrations (<400 ppb), the response of the developed TiO_2_-PEDOT nanocables exhibits linear behavior (Figure 5B inset). The normalized resistance change could reach 0.14% upon 100 ppb NO_2_ exposure. The detection limit was estimated to be 7 ppb NO_2_ at a signal-to-noise ratio of 3, which is lower than EPA current standard of 53 ppb NO_2_ for an annual arithmetic mean [34]. However, in the high range of NO_2_ concentration (0.4–85 ppm), another linear region was observed with less sensitivity (slope) in Figure 5B. There are only few reports using PEDOT as sensing materials in NO_2_ detection. Therefore, these results indicate that core-sheath TiO_2_-PEDOT nanocables are promising in sensitive NO_2_ detection. On the other hand, the normalized resistance change of TiO_2_-PEDOT nanofibers for NH_3_ shows a saturation behavior (Figure 6B). One can see that the response initially increases with the concentration of NH_3_ and then gradually levels off at a higher concentration. The detection limit was estimated to be 675 ppb NH_3_ (S/N = 3), which is significantly lower than the recommended threshold limit value of 25 ppm for human exposure [35] and better than most of reported values based on conducting polymers [19]. It is necessary to point out that the response magnitude of the TiO_2_-PEDOT nanocables for the same concentration of NH_3_ and/or NO_2_ is lower than those of PEDOT Langmuir-Blodgett (LB) film and PEDOT nanorods [15,16], however, the limits of detection for NO_2_ and NH_3_ achieved in this study is better than most reported values using PEDOT as the sensing element. As the limit of detection is dependent on response magnitude as well as the noise level, such good detection limit obtained in this study may be attributed to ultra-thin, homogeneous and uniform PEDOT layer, which can greatly suppress the noises generated from structural defects and thus improve the limit of detection.

## 3. Experimental Section

### 3.1. Materials

3,4-Ethylenedioxythiophene (EDOT), titanium isopropoxide (Ti(OiPr)_4_, 97%), and polyvinyl-pyrrolidone (PVP, MW = 1,300,000 g/mol) were obtained from Sigma–Aldrich. Acetic acid (99.8%) and ethanol were bought from Acros Organics and Fisher Scientific, respectively. For gas sensing studies, dry air, nitrogen dioxide (NO_2_, 1.036 × 10^4^ ppm and 49.28 ppm in dry air), and ammonia (NH_3_, 915.3 ppm in dry air) were purchased from Airgas.

### 3.2. Fabrication of TiO_2_ Nanofibers

TiO_2_ nanofibers were prepared by electrospinning using a well-established procedure [25]. Briefly, a 1 mL solution containing 0.1 mL Ti(OiPr)_4_, 0.2 mL acetic acid, 0.03g PVP and 0.7 mL ethanol was prepared and electrospun using a 23 gauge needle with a flow rate of 0.3 mL/h at an applied potential of 7 kV and a collection distance of 5 cm. The collected nanofibers were left in air overnight, followed by calcination in a muffle furnace at 500 °C for 3 h to remove PVP and generate TiO_2_ nanofibers.

### 3.3. Synthesis of Conductive Core-sheath TiO_2_-PEDOT Nanocables

Vapor deposition polymerization was employed to synthesize the ultra-thin PEDOT coating layer. The collected TiO_2_ nanofibers serving as the template for PEDOT coating was carefully dipped into 0.1 mol/L FeCl_3_ ethanol solution for 30 min, and then taken out and dried in air for 10 min. After the TiO_2_ nanofibers with adsorbed FeCl_3_ were exposed to saturated EDOT vapor at 95 °C for 2 days, TiO_2_-PEDOT nanocables were produced with TiO_2_ as the core and PEDOT as the sheath.

### 3.4. Characterization of TiO_2_ Nanofibers and TiO_2_-PEDOT Nanocables

Field emission scanning electron microscopy (FESEM) and transmission electron microscopy (TEM) were employed to characterize the morphology and the size of the as-prepared TiO_2_ nanofibers and TiO_2_-PEDOT nanocables. An attached Oxford energy dispersive X-ray (EDX) detector in the FESEM was used for chemical composition analysis. Fourier Transform Infrared (FT-IR) spectra were recorded for KBr disks containing samples with a Nicolet Magna-IR 560 spectrometer. The IR spectra were analyzed using the software of Omnic 7.2a from Thermo Electron Corporation. X-ray diffraction patterns (XRD) were obtained with an Oxford diffraction XcaliburTM PX Ultra with ONYX detector to study the crystal structure of the samples. The thermogravimetry (TG) was performed using a TA instruments (TGA Q500) under an air flow of 60 mL/min and a heating rate of 10 °C/min from room temperature to 800 °C.

### 3.5. Gas Sensing

A simple TiO_2_-PEDOT nanocables-based resistor-type gas sensor was fabricated by placing two electrodes on the as-prepared nanocables with a gap of ∼1 mm. Gas sensing experiments were carried out in a homemade 5 cm^3^ sealed glass chamber with gas inlet and outlet ports. The sensor circuit was subjected to a fixed 0.1 V DC bias and the current was continuously measured by a CHI-601C electrochemical analyzer (CH Instruments Inc., Austin, TX, USA). Dry air was used as carrier gas to obtain the diluted analyte mixture. The mixing of the analyte and carrier gas was regulated by computer-controlled gas mixing system (S-4000, Environics Inc., Tolland, CT, USA). The total flow rate of the gas mixture was set to be 1.2 L/min for NO_2_ detection and 0.2 L/min for NH_3_ sensing. Prior to the measurement, a stable baseline was obtained by purging dry air through the sensor for one hour, and then NO_2_ and NH_3_ with various concentrations was introduced to the gas chamber. In a typical NO_2_ sensing experiment, the sensor was first exposed to NO_2_ (100 ppb to 85 ppm) for 5 min, followed by dry air for 15 min to recover the sensor, and then the procedure was repeated. For NH_3_ sensing (5 to 30 ppm), the similar procedure was applied except the exposure and recovery time is 15 min and 30 min, respectively. The electric resistance of the sensor was calculated by applying Ohm’s law (R = V/I) while the normalized resistance change is defined as ΔR/R_0_% = [(R – R_0_) / R_0_] × 100%, where R_0_ is the initial electrical resistance of the sensor in dry air and R is the measured real-time resistance. All experiments were conducted at ambient temperature.

## 4. Conclusions

In summary, we have demonstrated a simple and efficient approach to fabricate conductive core-sheath TiO_2_-PEDOT nanocables using the as-electrospun TiO_2_ nanofibers as template. The as-prepared TiO_2_-PEDOT nanocables possess highly porous structure, large surface to volume ratio, and ultra-thin PEDOT layer, providing an excellent material for gas sensing. When used in chemiresistive mode, the TiO_2_-PEDOT nanocables exhibited good, reversible, and reproducible concentration-dependent response to gaseous NH_3_ and NO_2_. The good limit of detection and fast response/recovery can be attributed to background noise reduction and free access of gaseous molecules to ultra-thin PEDOT. This study provides a promising route for the facile and cost-effective synthesis of conductive nanomaterial with ultrathin conducting PEDOT layer.
